# Efficiency of an intensive educational program for informal caregivers of hospitalized, dependent patients: cluster randomized trial

**DOI:** 10.1186/s12912-015-0055-0

**Published:** 2015-01-31

**Authors:** Ana Rodríguez-Gonzalo, Carlos García-Martí, Ascensión Ocaña-Colorado, M José Baquera-De Micheo, Silvia Morel-Fernández

**Affiliations:** Hospital Universitario Ramón y Cajal, Crta. Colmenar Viejo, Km. 9.100, Madrid 28034 Spain

**Keywords:** Health education, Care givers, Continuity of care, Dependent patients, Quality of life

## Abstract

**Background:**

Educational initiatives for informal caregivers have proved efficient at reducing some of their symptoms, consequence of their involvement in care giving. However, more progress must be made in terms of the design of more successful interventions. Aims: Randomized clinical trial to test the efficiency of an Education Program for Primary Informal Caregivers of Hospitalized Dependent Patients in relation to their burden, mental and physical health, and care related knowledge.

**Methods:**

Design: Cluster Randomized Trial. Sample: 151 participants, primary caregivers of hospitalized, dependent patients, carried out from February 2009 to March 2010. They were assigned at random to two groups: one received an intensive educational program (n = 78), and the other just a generic speech (n = 73). The degree of burden of caregivers was recorded (Zarit Test), as well as their physical and mental health (SF12) and their knowledge of caregiving, before, immediately, after and one and a half months after the intervention. These analyses were carried out according to the Generalized Estimated Equations Method, in order to assess any possible improvements.

**Results:**

Participants´ burden did not improve, as measured by Zarit Test (p = 0,338), nor did their physical (p = 0,917) or mental health (p = 0,345). However there was an improvement in their hygiene caregiving (p = 0,001) and mobility care giving (p = 0,001).

**Conclusions:**

Caregivers found useful the education program, providing them with an informal support group. Interventions need to be longer and more customized as well as adapted to specific demands. There is a lack of validated questionnaires to assess improvements in care knowledge. There is a need to develop programs that contemplate continuity of care from primary to specialized caregiving.

**Trial registration:**

Cluster randomized trial: ESCPD2010

## Background

Sociodemographic changes in Spain are causing a growth in dependent population. Over 65 and over 80 population has doubled in the last 30 and 20 years respectively [1,2]. Moreover, we must take into consideration dependence caused by other circumstances [1].

Despite institutional aid and other support services, large numbers of family members still provide care to dependent patients, mainly at home, where the main aspects of vital needs care takes place [3,4]. Personalized care to dependent patients is generally carried out by primary or informal caregivers [5,6], largely female (83,6%), and normally daughters/sons (57,2%) [7].

In this group of primary caregivers, depression, anxiety and stress are common problems, as well as lumbago, arthritis and hypertension, with consequences on the social and family spheres [8,9]. Institutional, professional, social and family support, as well as efficient coping strategies, can reduce these adverse effects [10].

In the context of specialized nursing, with the aim of providing training and support to caregivers and minimizing the negative impact of long-term caregiving, a number of interventions and support programs are being implemented [4].

Emotional and Education interventions aim to give people a chance to express fears, improve their caregiving skills and find other forms of support, while hoping to avoid caregivers´ burden and the potential institutionalization as a consequence [5].

However, it has been difficult to determine the degree of efficiency of such educational programs for primary caregivers, due to differences in methodology and content: these programs have been implemented by various support services and aid groups, with very different aims, contents and forms of action. Meta-analyses and systematic assessments carried out since the late 80s have been evolving: after an early period of serious doubts in terms of efficiency [11], a series of meta-analyses [12-14] began showing moderate positive effects in various dimensions, such as degree of burden, depression, subjective wellbeing, satisfaction with care, skills and knowledge.

It is still unclear which interventions are most effective for which informal caregivers. Our project intends to cast light into this area, increasing our knowledge of the efficiency of educational interventions.

## Methods

### Design

#### Aim

The aim was to assess whether nursing interventions based on Health Educational Programs for primary informal caregivers of dependent patients may improve their quality of life, decrease emotional burden and increase caregiving knowledge, in order to better meet patients’ basic needs.

#### Study design

Cluster Randomized trial, carried out between February 2009 and March 2010, at Hospital Ramón y Cajal (Madrid), integrated in the Spanish national hospital network.

#### Sample

Primary informal caregivers of dependent patients, admitted in 30 hospital units, with expected hospital stays of at least one week. A dependent patient is understood to require the aid of someone else in order to carry out basic daily activities.

Exclusion criteria: caregivers with cognitive deficits that could be an obstacle for adequate verbal understanding, illiteracy, persons taking part in other clinical trials or who had taken part in similar workshops in the previous two years, as well as health professionals.

#### Sample size

Sample calculations were carried out, in order to detect average differences of 5 points in the SF-12 mental health scale between the groups. For this purpose, with a DS = 10, a confidence level of 95% bilateral, power of 80% and an assessment of losses of 10%, 71 patients were required per branch. Being a clusters study, this figure needed to be multiplied by the Inflation Factor (IF). In this case, starting from an ICC = 0.01 (obtained from scientific literature) [15], and from an average cluster size of 4.3 (average of participants during pilot study, one month previous to actual study):

$$ FI = 1 + \left(\left( cluster\  size - 1\right)\ *\ ICC\right) = \Big(1 + \left(\left(4.3 - 1\right)\ *\ 0.01\right) = 1.033. $$

Thus, the size of each of the groups (intervention and control) needed to be of:

$$ 71\ *\ 1.033 = 73.3 = 73\  caregivers\  per\  branch $$

#### Instruments

- Quality of life assessed through SF12 questionnaire, both mental and physical health scales; response format were Likert scales of 3 or 5 points, to assess the intensity or frequency depending on the item. Test scores were obtained using algorithms developed by the authors, scores being normalized to a scale of 0 to 100, with 50 as the average scale of the normal population. It was a reduced SF36 questionnaire. The prediction capability of the SF36 results was a R^2^ of 0.911 and 0.918, for the Physical and Mental Health Scales respectively. The test-retest reliability after 2 weeks showed Pearson Coefficients of 0.89 for the Physical Health Scale and 0.76 for the Mental Health Scale [16]. In terms of relative validity, coefficients for the physical scale were 0.43 to 0.93 (average = 0.67) and for the mental scale 0.60 to 1.07 (average = 0.97). In terms of the reliability and validity of the Spanish version of the SF36, joint estimates obtained from meta-analyses [17] of α coefficients of Cronbach were ≥ 0.9 for the scales Physical function, Physical role and Emotional role, and the joint estimates of the rest of the scales were all above 0.7. In terms of the validity of the construct, it showed a correlation with the General Health Questionnaire (GHQ) [18], and the St. George’s Respiratory Questionnaire (SGRQ) [19].
- Caregiver´s burden assessed through Zarit´s Test, consisting of 22 items, responded according to Likert scale of 5 points. Scores result from adding the points obtained from all responses. In Spain two cutoff points have been established, one between “no burden” and “burden”; between 46 and 47 points; with an 84.4% specificity and 85.1% sensibility. The other, between “mild burden” and “severe burden”; between 55 and 56 points; with a 89.7% sensibility and 94.2% specificity; an internal consistency of Cronbach alfa coefficient of 0.91%; and a test-retest reliability after 3 months of Pearson coefficient of 0.86 [20].
- Caregiving knowledge before and after the intervention, assessed through a self-designed questionnaire consisting of 5 items: feeding, elimination, mobility, hygiene and emotional self-care, with a response scale (Likert type) of 5 points. Global and specific knowledge of each item were analyzed. It showed an internal consistency Crombach alpha of 0,889.
- Patient degree of dependency, assessed through Katz Index, which has shown a good intra-observer reproducibility in all studies [21-23]. Correlation coefficients (r) between 0.73 and 0.988, with an adequate content and construct validity. It was used as gold standard to compare new functional assessment indexes [21,23].
- Socio-economic variables: both patient and caregiver age and gender, caregiver relation to the patient, educational level, work status, caregiving time, number of persons receiving care, primary or only caregiver, and hospitalized patient´s pathology.

#### Interventions

The intervention in the experimental group was an intensive educational program, organised and structured, which included two training sessions, taught consecutively on a weekly basis. Each session lasted 3 hours and was theoretical and practical in nature. The methodology involved active and dynamic participation. Caregivers had the opportunity to acquire a wide range of valuable knowledge through practice. The caregiver was trained in the skills necessary to carry out basic daily activities, in response to group demands, while encouraging emotional relief and offering emotional support through active listening.

The session started with the introduction of each participant stating their situation and particular needs. Then the training program would begin, divided in 5 areas but focusing on the specific demands of each group:

- Feeding: nutritional advice, adequate food handling, importance of adequate hydration and device handling.
- Disposal: constipation and diarrhea, signs of alarm, handling of nappies, devices, urinary catheters.
- Mobility: position change, prevention of falls, relation between bed rest and skin injuries, incorporation, aided mobilizations, ergonomics to prevent injuries in caregiver.
- Hygiene: bed rest, nails, head, mouth, creases and probes, relation to the prevention of skin injuries.
- Emotional self-care: removing culpability, emotional expression, time management, asking for help, social networks and search for support.

This training was complemented with an educational dossier, which was handed over to each participant, and which was published by a social foundation, with exhaustive information on the contents of the workshops [24].

The control group (GC) in turn received a single 2 hours session, which was also taught on a weekly basis, on generic life and aging processes.

#### Randomization

Participants were all primary caregivers of hospitalized dependent patients, sharing rooms and public spaces during their stay. Therefore, it was necessary to guarantee that experimental and control group participants would not meet. In order to avoid participants´ contamination, randomization was stratified by hospitalization units.

Randomization sequencing was carried out using Epidata computer program. A person external to the study generates two sequences, one for the type of intervention and the other for the units in which it would be recruited every week. She then handed them over to the study team in closed, numbered envelops, each had to be opened in the sequential order of the week.

#### Ethical considerations

The study was approved by the hospital Research Ethics Committee, “*Research Ethics Committee Hospital Ramón y Cajal”.*

### Data collection

#### Simple blind design

The lead investigator was the only person in charge of safeguarding and opening the envelops. Once she had found out the units where recruitment would take place, she informed the team in charge, who ignored to which group patients were being assigned: this team, formed by two persons, then proceeded to the selected units, where it was decided, together with the unit nursing team, which patients satisfied inclusion criteria. Caregivers were then offered to take part in information sessions for patients´ family members, and they were handed over written information on the study, as well as informed consent approved by the hospital Ethics Committee. Once they agreed to participate they were handed over the questionnaires referred above. The nurse would then complete the Katz Index of the hospitalized patient.

When the recruitment process was over, one day before the first session, the envelop with the type of intervention, either experimental or control, was opened in order to inform the team responsible for carrying it out. The day of the intervention a member of the team who had not taken part in the recruitment process, located the participants and led them to the class room.

Three to 4 days after the intervention and before their discharge, they would again complete the SF12, the Zarit´s Questionnaire and the skills knowledge questionnaire.

Finally, one month later and back home, they were requested to respond again, through postal mail, to the SF12 and Zarit´s Questionnaire, and the Katz Index of the patient was assessed one again through the phone.

### Data analysis

Data analysis was conducted with SPSS 15.0 for descriptive and correlation analyses, and with STATA for multivariate causal analyses. All analyses were conducted assuming a confidence level of 95%.

Descriptive analysis began by testing the normality of scores with Kolmogorov-Smirnov Test, and inter-group homogeneity with Chi Squared Test. Standard deviation and average of the variables that met the normality criteria were calculated. Otherwise the choice was the interquartile range and the median. In turn, nominal variables were analyzed by frequency and percentage.

Once the descriptive analysis was finished, the correlation analysis was conducted. Different tests were used for this purpose, depending on the characteristics of the variables. For two continuous variables Pearson Correlation was used, as long as both satisfied normality criteria. Otherwise it was Spearman correlation. For nominal variables, Chi squared test was used. Finally, correlations between nominal and continuous variables were analysed using two different tests depending on the characteristics of the nominal variable: if it included more than two categories the choice was ANOVA test; if dichotomic, Student´s T test for average comparisons.

Multivariate causal analysis was conducted in order to find out whether the intervention had modified the caregiver´s quality of life (mental and physical SF12 scales), burden (Zarit’s Burden Index) or caregiving knowledge (self-designed questionnaire). Since the randomization was conducted through hospitalization units and not individuals, it was mandatory to analyze each week’s education group as a unit, and therefore to carry out a cluster analysis where each cluster corresponded to each week’s education group. For this purpose the Generalized Estimated Equations technique available in STATA statistical package was employed.

A maximum model was designed which included the variables that had shown the greatest correlation with the dependent variables: type of intervention, Katz Index, Relation with the Patient, Caregiver Gender, Caregiver Age, Caregiving Time, Family Loads and Educational Level. To this model was added, for each particular case, the results of the dependent variable before the intervention, up to a total of 9 variables.

Once the maximum model was defined, nominal variables of more than two categories (Caregiving Time and Educational Level) were transformed into binomial variables in order to improve the reliability of results, since GEE´s analysis better functions as the number of empty or small size responses decreases.

After the multinomial analysis an Intention to Treat Analysis (ITA) for lost values was conducted, as recommended by CONSORT protocol [25] for Randomized Clinical Tests. Among the various existing criteria for value assignment, we choose to assume the worst possible scenario, since it is the most extensively used [26,27]. In this strategy, the control group lost values are substituted by the experimental group best score, and the experimental group lost values by the control group worst score.

## Results

### Participant flow

**Figure 1:**
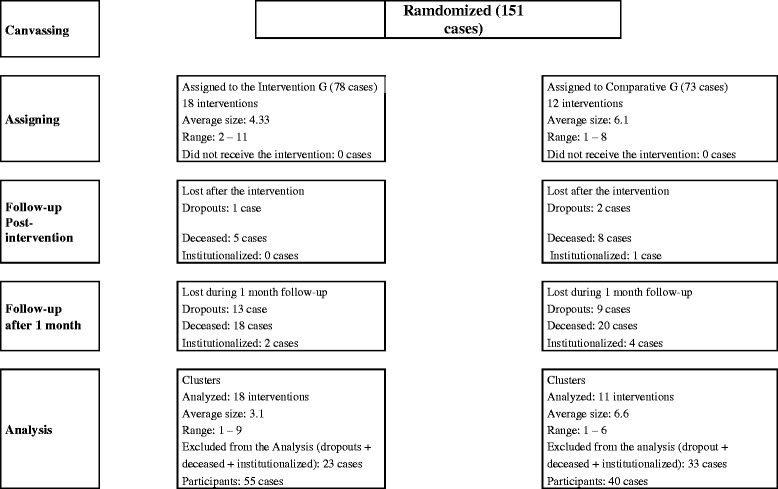
**Randomization and Participant Flow.**

Here is the research's participant flow (Figure 1).

#### Baseline data

151 participants were recruited. Kolmogorov-Smirnov test certified that variables were normally distributed, except for participants´ age. It was subsequently certified that there was no significant differences between the experimental and the control group.

Patients were of old age (average 80 years old, 71–86), with a similar gender distribution, 80 women (53%), with heterogeneous pathologies. Most came from Internal Medicine Units (n = 60, 40%), with a severe dependency, (n = 126, 83.4%), and most were dependent before being hospitalized (n = 96, 63.6%, Table 1).

**Table 1:** **Characteristics of patients**

**Characteristics of subjects**			**Experimental (N = 78)**	**Control (N = 73)**
Patients				
	Age	(median, interquartile range)	7.5 (70.5 - 87.25)	82 (71–86)
	Sex	Female	45/78 (58) (47–69)	35/73 (48) (37–59)
	Main diagnosis			
		Internal medicine, n/N (%) (I.C. %)	35/78 (45) (34–56)	25/73 (34) (23–45)
		Oncology, n/N (% )(I.C. %)	17/78 (22) (13–31)	20/73 (27) (17–37)
		Neurology, n/N (%) (I.C. %)	8/78 (10) (3 – 17)	5/73 (7) (1–13)
		Vascular, n/N (%) (I.C. %)	7/78 (9) (3–15)	4/73 (6) (1–11)
		Trauma, n/N (%) (I.C. %)	7/78 (9) (3–15)	11/73 (15) (7–23)
		Cardiology, n/N (%) (I.C. %)	2/78 (3) (1–7)	2/73 (3) (0–7)
		NS/NC, n/N (%) (I.C. %)	2/78 (3) (1–7)	6/73 (8) (2–14)
	Katz Index			
		Severe Dependency, n/N (%) (I.C. %)	68/78 (87) (80–94)	59/73 (81) (72–90)
		Moderate Dependency, n/N (%) (I.C. %)	7/78 (9) (3–15)	11/73 (15) (4–18)
		No Dependency, n/N (%) (I.C. %)	3/78 (4) (0–8)	3/73 (4) (0–9)
	Previous Dependency			
		Yes, n/N (%) (I.C. %)	56/78 (72) (62–82)	40/73 (55) (44–66)
		No, n/N (%) (I.C. %)	19/78 (24) (15–35)	28/73 (38) (27–49)
		DK/NA/ REF, n/N (%) (I.C. %)	3/78 (4) (0–8)	5/73 (7) (1–13)

%: percent.

I.C.: confidence interval.

N: sample size.

Caregivers, in turn, were mostly women (n = 121, 80%). Average age was around 50 (53.8, D.T. 15.3). Most caregivers were sons/daughters (n = 55, 36.4%) and partners (n = 37, 24.5%). 45.7% were unemployed. 45% had been caregiving their family member for over one year (Table 2). Table 3 shows characteristics per cluster and Table 4 shows the dependet variables results.

**Table 2:** **Characteristics of caregivers**

**Characteristics of subjects**			**Experimental (N = 78)**	**Control (N = 73)**
Caregivers				
	Age		54.8 (15.1)	52.7 (15.5)
	Sex	Female	62/78 (80) (71–89)	59/73 (81) (72–90)
	Relation to Patient			
		Partner, n/N (%) (I.C. %)	21/78 (27) (17–37)	16/73 (22) (13–32)
		Son/Daughter, n/N (%) (I.C. %)	27/78 (35) (24–46)	33/73 (45) (34–56)
		Brother/Sister, n/N (%) (I.C. %)	4/78 (5) (0–10)	4/73 (6) (1–11)
		Parent, n/N (%) (I.C. %)	2/78 (3) (0–7)	3/73 (4) (0–9)
		Professional Caregiver, n/N (%) (I.C. %)	14/78 (18) (9–27)	10/73 (14) (6–22)
		Other, n/N (%) (I.C. %)	10/78 (13) (6–20)	7/73 (10) (3–17)
	Educational Level			
		Read and Write, n/N (%) (I.C. %)	9/78 (12) (5–19)	12/73 (16) (8–24)
		Primary Education, n/N (%) (I.C. %)	21/78 (27) (17–37)	14/73 (19) (10–28)
		Secondary Education, n/N (%) (I.C. %)	12/78 (15) (7–23)	12/73 (16) (8–24)
		O - Levels and A - Levels, n/N (%) (I.C. %)	14/78 (18) (9–27)	24/73 (33) (22–44)
		University Education, n/N (%) (I.C. %)	20/78 (26) (16–36)	11/73 (15) (7–23)
		DK /NA/REF, n/N (%) (I.C. %)	2/78 (3) (0–7)	0/73 (0)
	Work Situation			
		Active, n/N (%) (I.C. %)	30/78 (39) (28–50)	36/73 (43) (32–54)
		Unemployed, n/N (%) (I.C. %)	8/78 (10) (3–17)	7/73 (10) (3–17)
		Inactive, n/N (%) (I.C. %)	39/78 (50) (39–61)	30/73 (41) (30–52)
		DK/NA/REF, n/N (%) (I.C. %)	1/78 (1) (0–3)	0/73 (0)
	Caregiving Time			
		None, n/N (%) (I.C. %)	4/78 (5) (0–10)	10/73 (14) (6–22)
		0 - 6 Months, n/N (%) (I.C. %)	29/78 (37) (26–48)	27/73 (37) (26–48)
		6 - 12 Months, n/N (%) (I.C. %)	5/78 (6) (1–11)	5/73 (7) (1–13)
		>12 Months, n/N (%) (I.C. %)	40/78 (51) (40–62)	28/73 (38) (27–49)
	Family Extra Loads			
		0, n/N (%) (I.C. %)	17/78 (22) (13–31)	20/73 (27) (17–37)
		1, n/N (%) (I.C. %)	41/78 (53) (42–64)	29/73 (40) (29–51)
		2, n/N (%) (I.C. %)	7/78 (9) (3–15)	13/73 (18) ( 9–27)
		3, n/N (%) (I.C. %)	5/78 (6) (1–11)	3/73 (4) (0–9)
		>3, n/N (%) (I.C. %)	1/78 (1) (0–3)	2/73 (3) (0–7)
		DK/NA/REF, n/N (%) (I.C. %)	7/78 (9) (3–15)	6/73 (8) (2–14)
	Type of Caregiver			
		Single, n/N (%) (I.C. %)	36/78 (46) (35–57)	34/73 (47) (36–58)
		Primary, n/N (%) (I.C. %)	38/78 (49) (38–60)	39/73 (53) (42–64)
		DK/ NA/ REF, n/N (%) (I.C. %)	4/78 (5) (0–10)	0 (0)

%: percent I.C.: confidence interval N: sample size.

**Table 3:** **Characteristics of patients and caregivers per cluster**

**Characteristics clusters**			**Experimental (n = 12)**	**Control (n = 18)**
**Size, average (D.S.)**			**6.5 (2.65)**	**4.1 (1.89)**
Patients				
	Age (average, DS)		75.3 (7.41)	77,5 (7.58)
	Gender	Female, (%)	58	40
	Primary Diagnosis	Internal Medicine, (%)	46	40
		Oncology, (%)	21	24
		Neurology, (%)	11	6
		Vascular, (%)	7	6
		Trauma., (%)	8	13
		Cardiology, (%)	3	4
		DK/NA/REF, (%)	3	6
	Katz Index	Severe Dependency, (%)	87	84
		Moderate Dependency, (%)	8	13
		No Dependency, (%)	4	3
	Dependency Previous to Hospitalization	Yes, %	73	73
		No, %	23	23
		DK/NA/REF, %	5	5
Caregivers				
	Age (average, DS)		56.1 (7.99)	51.7 (8.15)
	Gender	Female, (%)	78	76
	Relation to Patient	Partner, (%)	29	20
		Son/Daughter, (%)	37	46
		Brother/Sister, (%)	6	5
		Parent, (%)	2	6
		Professional Caregiver (%)	14	13
		Others, (%)	12	10
	Educational Level	Read and Write, (%)	15	14
		Primary Education, (%)	26	22
		Secondary Education, (%)	15	18
		O – Levels and A - Levels, (%)	16	32
		University Education, (%)	28	15
	Work Situation	Active, (%)	35	42
		Unemployed, (%)	12	13
		Inactive, (%)	52	45
	Caregiving Time	None, (%)	5	12
		0 - 6 Months, (%)	35	37
		6 - 12 Months, (%)	5	8
		>12 Months, (%)	55	40
		DK/NA/REF, (%)	0	3
	Family Extra Loads	1 person (%)	75	62
		More than 1 person (%)	15	31
		DK/NA/REF, (%)	9	7
	Type of Caregiver	Single, (%)	45	42
		Primary, (%)	51	58
		DK/NA/REF, (%)	4	0

%: percent.

n: sample size.

D.S.: standar deviation.

**Table 4:** **Analyzed dependent variables**

**Dependent variables**		**Experimental group**		
		**instant 0**	**Instant 1**	**Instant 2**
		**Average (D.S.)**	**Average (D.S.)**	**Average (D.S.)**
Instrument				
SF12	Physical health Scale	45.9 (11.17)	44.4 (11.54)	45.6 (8.99)
	Mental Health Scale	45.2 (10.51)	44.5 (11.80)	46.5 (11.85)
Zarit Overload		2.7 (0.71)	2.5 ( (0.89)	2.6 (0.84)
Knowledge Questionnaire				
	Mobility Knowledge, average (D.S.)	1.8 (1.23)	3.3 (0.87)	
	Feeding Knowledge, average (D.S.)	2.6 (1.29)	3.3 (0.89)	
	Disposal Knowledge, average (D.S.)	2.2 (1.39)	3.3 (0.71)	
	Hygiene Knowledge, average (D.S.)	2.7 (1.19)	3.5 (0.85)	
	Emotional Expression Knowledge, average (D.S.)	2.3 (1.34)	3.2 (0.99)	
	average (D.S.)Average Knowledge,	2.3 (1.06)	3.3 (0.76)	
**Dependent variable**		**Control group**		
		**Instant 0**	**Instant 1**	**Instant 2**
		**Average (D.S.)**	**Average (D.S.)**	**Average (D.S.)**
Instrument				
SF12	Physical Health Scale	47.1 (9.78)	47.7 (10.60)	47 (9.99)
	Mental Health Scale	43.2 (13.02)	42.9 (13.17)	44 (13.85)
Zarit Overload		2.5 (0.77)	2.3 (0.97)	2.4 (0.72)
Kowledge Questionnaire	Mobility Knowledge, average (D.S.)	2.1 (1.53)	2.6 (1.26)	
	Feeding Knowledge, average (D.S.)	2.5 (1.37)	3.2 (0.99)	
	Disposal Knowledge, average (D.S.)	2 (1.47)	3 (1.09)	
	Hygiene Knowledge, average (D.S.)	2.8 (1.41)	3.2 (1.11)	
	Emotional self-care, average (D.S.)	2.5 (1.41)	3 (1.02)	
	Average Knowledge, average (D.S.)	2.4 (1.17)	3 (0.91)	

Descriptive Results.

D.S.: standar deviation.

### Numbers analyzed

78 participants began the study in the intervention group and 73 in the control group. In the experimental group there were 18 losses due to death, 2 due to institutionalization and 3 abandonments, so the analysis was conducted on 55 cases (55/78). In the control group there were 20 deaths, 4 institutionalizations and 9 abandonments, so the analysis sample was conducted on 40 cases (40/73).

### Multinomial analysis

Next the multinomial analysis was conducted (Table 5). The evolution from before the intervention to immediately after (instants 0 and 1) contradicted our hypothesis for physical health: it worsened in the intervention group and slightly improved in the control group, showing a statistically significant development, only negative (coefficient −2.46, p = 0.009). In the other dimensions there was no significant change, not for the Mental Health Index (p = 0.589), nor for Zarit Burden (p = 0.281). Results were similar after conducting a univariate analysis considering just the type of intervention: the effect on Physical Health was −2.77 (p = 0.003) and there was no significant effect on Mental Health nor on Zarit Burden (p = 0.123).

**Table 5:** **Multinomial analysis, causation analysis**

			**GEE instant 0 a 1 univariate**	**GEE Instant 0 to 2 multivariant**	**GEE instant 0 to 2 univariate**	**AIT**
		**Coefficient (intervals) (p)**	**Coefficient (intervals) (p)**	**Coefficient (intervals) (p)**	**Coefficient (intervals) (p)**	**Coefficient (intervals) (p)**
Instrument						
SF12	Physical Health Scale	−2.46 (−4,31 – −0.61) *(p = 0,009)	−2,77 (−4,58 – −0,97) *(p = 0,003)	−0,19 (−3.65 - 3,28) (p = 0,917)	0,10 (−2,93 - 3,14) (p = 0,946)	
	Mental Health Scale	1.06 (−2.79 – 4.92) (p = 0.589)	0.45 (−3.22 – 4.12) (p = 0.810)	2.89 (−2.27 – 8.05) (p = 0.272)	2.56 (−2.75 – 7.88) (p = 0.345)	
Zarit Overload		0,16 (−0.13 – 0.46) (p = 0.281)	0.24 (−0.07 – 0.55) (p = 0.123)	0,16 (−0.16 – 4.73) (p = 0.338)	0.11 (−0.22 – 0.44) (p = 0.523)	
Knowledge Questionnaire	Mobility Knowledge, average (D.E.)	0.78 (0.38 – 1.18) *(p = 0.001)				0.39 (−0,10 – 0.89) (p = 0.119)
	Feeding Knowledge, average (D.E.)	0.13 (−0.14 – 0.41) (p = 0.329)				
	Disposal Knowledge, maverage (D.E.)	0,14 (−0.17 – 0.46) (p = 0.373)				
	Hygiene Knowledge, average (D.E.)	0.40 (0.20 – 0.60) *(p = 0.001)				0.12 (−0.20 - 0,44) (p = 0.470)
	Emotional self-care, average (D.E.)	0,22 (−0,06 - 0,49) (p = 0.119)				
	Average Knowledge, Average (D.E.)	0,27 (−0,04 - 0,59) (p = 0.089)				

Note: *statistically significant results.

In terms of the total evolution, this is, from recruitment to one month after the intervention (instant 0 to 2), there was no significant effects for the Physical Health Index (p = 0.345) of this questionnaire; nor for Burden according to Zarit Test (p = 0.338). This situation did not change either when considering just the type of intervention and the initial score, with p = 0.946 for the Physical Health Index, p = 0.272 for the Mental Health Index and p = 0.523 for the Zarit Burden Index.

In terms of acquired knowledge, the intervention participants improved their mobility knowledge by 0.77 points in a 0 to 4 scale as a result of taking part in the experimental workshop (p = 0.001), and 0,39 points in hygiene (p = 0.001). However, other variables did not show significant progress: the improvement in feeding knowledge of 0.13 points did not reach statistical significance (p = 0.329); nor did the 0.14 points improvement in disposal knowledge (p = 0.373); or the 0.21 in Emotional self-care (p = 0.119). All together, the average improvement in knowledge of 0.27 points was not significant either (p = 0.089).

### Intention to treat analysis

Multinomial analysis conclusions indicated that despite no significant improvement on Quality of Life or Burden had been achieved, significant progress had been achieved in Caregiving Knowledge.

Once the lost values had been substituted following these criteria, the ITA was conducted. In this case only the initial scores in the respective dimensions were included: mobility and hygiene knowledge declared before the intervention. The absence of other possible confounder and interaction variables was decided in order to insure the statistical power of the test. This ITA result was the loss of significance of the improvements in mobility (0.39, p = 0.119) and hygiene (0.12, p = 0.470).

### Intraclusters correlation coefficients

As part of the analyses and following CONSORT declaration recommendations [25], Intracluster Correlation Coefficients (CCI) of the final values were calculated, so that we could contribute to sample size calculations of future investigations. Table 6 shows these results.

**Table 6:** **Intracluster correlation coefficients**

**Variable**	**Intracluster Correlation Coefficient (ICC)**
Katz Index Instant 2	0.0002
Physical Scale SF12 Instant 2	0.0026
Mental Scale SF12 Instant 2	0.0046
Zarit Overload Instant 2	0.0032

## Discussion

Our research hypothesis was that hospitalized, dependent patients´ caregiver´s participation in a Health Educational Program could improve their quality of life, and reduce perceived burden, as well as improve caregiving knowledge. The statistical analysis results showed that no significant improvement in the two main variables was recorded. This outcome partially rejects our initial hypothesis. However, our intervention managed to increase the caregiving knowledge of caregivers, in terms of mobility and hygiene.

These results must be contextualized along collected evidence on these kinds of interventions since the 1980´s. Two successive meta-analyses by Pinquart [14] and Sorensen [13] found moderate positive effects in several dimensions: caregiving knowledge, burden, depressive symptoms, personal wellbeing and work satisfaction. Among these, only caregiving knowledge showed a medium size effect, while the other variables showed a significant but small effect. Also, these effects depended on the kind of intervention carried out, with psychoeducational and psychotherapeutic interventions showing the most consistent effects, above those of respite care, support groups, knowledge and multicomponent.

In turn, meta-analysis conducted by Thompson *et al.* in [28] only found significant improvements on depressive symptoms and for group psychoeducational interventions, while the effects of the rest of interventions and on the rest of the variables (burden, quality of life, etc.) were not significant.

On balance, the three meta-analyses referred above prove moderate effects on certain variables and on certain type of interventions. This conclusion is similar to those reached by previous meta-analyses, such as those by Knight *et al.* [12], that found larger effects in individual interventions than in group interventions, and a very low effect in respite interventions; Acton and Kang [29], that only identified improvements in burden in multicomponent interventions; or Brodaty *et al.* [30], that found significant improvements on emotional stress and knowledge, but not on burden, and systematic revisions such as those by Schulz *et al.* en [31], with small or moderate effects on burden, psychological symptoms and quality of life; Lee and Cameron [32], that did not find effects in respite interventions, and Garcés *et al.* [33], that found contradictory results both in respite and psychoeducational interventions, with interventions showing positive effects and others no effects.

Thus, literature confirms the great difficulty in conducting effective interventions. Effectivity depends on multiple factors: type of intervention, duration, type of target population, recruitment and randomization strategies, aims, measured variables, etc. Accordingly, our study’s moderate effects have been similar to those of previously published interventions.

There are two factors that may have contributed to the reduced effects of our intervention. Firstly, the recorded mortality: 25.2%. The diversity of pathologies of our participants makes comparisons difficult, but research by Rodgers *et al.* [34], in a hospital context and with stroke patients, also with a high mortality, showed just 12.7% mortality, which illustrates how high our rate was. This higher than expected mortality couldn’t be included during sample size calculation since the bibliography [34] taken into account for this operation didn’t reflect such elevated numbers, resulting in a reduced statistical power.

Moreover, mean differences found in the SF12 mental scale are smaller than what was initially estimated during sample size calculation. This, added to the high mortality found, doesn’t allow us to clearly determine the program effectivity.

Along with these statistical problems, we may talk about several limitations in this study. Burden and quality of life below average were not established as inclusion criteria, which might have limited the potential benefits of the intervention. In contrast to this decision, research found by Zarit and Femia [35] that determined a minimum of emotional stress for inclusion, obtained significant improvements [36-38].

Also, the necessary standardization of the intervention, even adapting to the needs of caregivers after the initial exploration of their demands, may have limited the suitability of contents, given the sociodemographic heterogeneity of caregivers’ profiles, which, nevertheless, matched their general population [39-42].

Another element to consider was the fact that the duration of the intervention may have not been sufficient to achieve significant improvement. However, it was unfeasible to increase intervention time. Accordingly, the meta-analysis of Sorensen y Pinquart [13] found significant differences in the achieved effect on burden, depressive symptoms and symptoms of the patients depending on the duration of the intervention. The same conclusion was reached by Zarit and Femia [35], this is, that longer studies [43-45] had significant effects hard to replicate by shorter ones [46-49].

Finally, we must refer to the improvement in the physical health state of the control group after the intervention, and the worsening of the intervention group. This was an unexpected effect of the intervention, which leads us to wonder whether attending the knowledge workshops may have caused an increased conscience of the caregiver of his/her role, which manifests in somatization and worsening of his/her perceived physical health, while the unexpected effect on the control group is one of a greater calm and tranquility. It is also worth reflecting on the instruments employed. Zarit Burden Questionnaire, although adequate to diagnose and measure burden, is not so for measuring intervention results, for some of their dimensions do not match fixed stressors that are difficult to modify. [12,50,51]. Moreover, the caregiver evolution was not controlled within the possible variations of their social support.

Also SF12 questionnaire, as most of HRQoL ones, has a low sensitivity towards change. Therefore, it would have been more appropriate to take more measures extended in time to check the intervention effects. This reduced version is also less sensible than the original SF36, however it was chosen precisely because of its shortness, given the caregivers lack of time and emotional stress in the hospitalization context.

On the other hand, those dimensions that we are able to modify, such as caregiving knowledge, lack validated questionnaires, which impedes gaining further knowledge and makes it necessary to employ indirect measures such as patients´ readmissions or life expectancies.

To conclude, we believe it is necessary to raise a debate on the suitability of the Randomized Clinical Trial for this kind of interventions. The possible inadequacy of Randomized Clinical Trials for psychosocial or educational interventions was demonstrated by Zarit [52] or Acton and Kang [29], and even some authors such as Dowling and Wiener [53], consider a moral dilemma to conduct interventions that lack any benefits (control group) on stressed and burdened caregivers, highly guilt ridden for abandoning their family member. The problems, as we see them, are of a practical nature too. Since participants recruitment becomes much more difficult, perhaps introducing bias into the psychosocial profile of participants, by encouraging the participation of caregivers with better resources and availability. The greatest quality of evidence can only be obtained through randomized clinical trials, but this implies a moral debate and leads us to reflect on the extent that research based on quasi-experimental pre-post designs ought to be employed.

We also need to refer to the results of the Intention to Treat Analysis. By adopting the worst possible scenario illogical effects occur: intervention group participants, by receiving the worst possible score, lose caregiving knowledge as a result of attending the speech. On the other hand, those in the control group, obtaining the best possible score, gain knowledge. It is clear how unreal this results are, so the strategy of adopting the worst possible scenario might not be suitable for this and other similar contexts.

## Conclusions

It was shown that the caregiving relation is a complex one, which varies from one individual to the next, and for this reason the intervention must be personalized and adapted as much as possible. It was also shown that it is a fixed stressors relation, and difficult to modify. All this is in accordance with previous literature as well as with our theoretical framework. However, as we have previously discussed, our intervention had several limitations, and was not as adjusted, flexible and long as it would have been desirable.

We believe there are two different debates in relation to caregivers training. On the one hand, if evidence shows that caregiving knowledge might not improve the caregiver´s quality of life, this does not imply that the caregiver does not have the right to receive such training. It might be the case that quality of life does not depend on knowledge, but patients Access to knowledge is independent of their quality of life, for it constitutes one of the strategic axes of our health system, which aims to confer the patient a central and active role.

On the other hand, there is no doubt that the aim ought to be to develop the most effective programs in order to improve the quality of life of patients and their caregivers. Accordingly, we believe that although the need for training and support in the hospital environment was adequately identified by our team, the efficiency of the programs can only be improved through continual care, involving a wide network of formal and informal caregivers. Thus, one of the future challenges is for primary and secondary care service units to develop joint programs, in order to achieve that caregivers can feel supported throughout the entire caregiving process.
